# Food ladder use among ASCIA-affiliated clinicians in Australia and New Zealand: an exploratory descriptive survey

**DOI:** 10.3389/falgy.2026.1889636

**Published:** 2026-07-28

**Authors:** Alan Nguyen, Yiran Tu, Kuang-Chih Hsiao, Jane Peake, Alberto Pinzon-Charry

**Affiliations:** 1 Epworth Allergy Specialists, Epworth HealthCare, Richmond, VIC, Australia; 2 Population Allergy, Murdoch Children's Research Institute, Parkville, VIC, Australia; 3 Department of Paediatrics, The University of Melbourne, Parkville, VIC, Australia; 4 Queensland Paediatric Immunology and Allergy Service, Queensland Children's Hospital, South Brisbane, QLD, Australia; 5 Department of Immunology, Starship Child Health, Te Whatu Ora - Health New Zealand, Auckland, New Zealand; 6 Department of Paediatrics: Child and Youth Health, University of Auckland, Auckland, New Zealand; 7 University of Queensland, St Lucia, QLD, Australia; 8 Griffith University, Nathan, QLD, Australia

**Keywords:** ASCIA, egg ladder, food ladders, IgE-mediated food allergy, milk ladder, non-IgE-mediated food allergy, paediatrics, survey

## Abstract

**Background:**

Food ladders are clinician-guided graded dietary advancement tools used in selected patients with food allergy, but real-world implementation in Australia and New Zealand is not well described.

**Objective:**

To characterise respondent-reported food ladder use and implementation patterns among ASCIA-affiliated clinicians in Australia and New Zealand.

**Method:**

We conducted a cross-sectional online survey of ASCIA-affiliated healthcare professionals from 1 to 30 June 2024. Invitations were distributed via the ASCIA email distribution list. Analyses were descriptive, with percentages calculated using valid-response denominators.

**Results:**

Seventy clinicians participated: Australia 45/70 (64.3%) and New Zealand 25/70 (35.7%). Disciplines included allergy/immunology specialists (29/70, 41.4%), general paediatricians (13/70, 18.6%), nurses/nurse practitioners (10/70, 14.3%), dietitians (10/70, 14.3%), trainee doctors (7/70, 10.0%), and one general practitioner (1/70, 1.4%). Most primarily saw paediatric patients (53/70, 75.7%). Food ladder use was commonly reported among respondents (66/70, 94.3%). Among ladder users, most reported use in both IgE- and non-IgE-mediated contexts (47/66, 71.2%); milk (65/66, 98.5%) and egg (64/66, 97.0%) ladders predominated. Standardised ladders, recipes, and home introduction protocols were reported by 43/66 (65.2%), 43/66 (65.2%), and 45/66 (68.2%) respondents, respectively. Prior anaphylaxis to the proposed ladder food (51/66, 77.3%) and poorly controlled asthma (38/66, 57.6%) were the most frequently cited contraindications. In scenario responses, clinicians were least likely to recommend a ladder when there was prior anaphylaxis to the relevant food: milk 9/70 (12.9%) and egg 13/70 (18.6%).

**Conclusion:**

Among respondents, food ladder use was commonly reported, particularly for milk and egg allergy, with variability in implementation supports and in responses to higher-risk scenarios. These exploratory, self-reported findings are descriptive and hypothesis-generating. Together, they encourage further prospective evaluation and consensus-based standardisation.

## Introduction

Food allergy contributes substantially to healthcare morbidity and resource utilisation. International cohort studies have reported challenge-proven cow's milk (hereafter milk) and hen's egg (hereafter egg) allergy in early childhood, and Australian population data from the HealthNuts cohort have highlighted a particularly high burden of challenge-confirmed food allergy in infancy (1–6). Food allergy management can also affect family quality of life through dietary restriction, uncertainty about reactions, and the need for emergency preparedness (7, 8).

Management of food allergy has evolved from strict avoidance alone towards more individualised approaches that may include supervised oral food challenges, dietary expansion where tolerated, and, in selected settings, immunomodulatory treatment. Food ladders are one such approach. They are clinician-guided graded dietary advancement tools that typically begin with extensively heated forms, such as baked milk or baked egg, and progress towards less heated forms where clinically appropriate. They should be distinguished from oral immunotherapy, which has different aims, dosing structures, monitoring requirements, and risk profiles.

Milk and egg ladders are the most established in clinical resources and published literature, whereas the evidence base for soy and wheat ladders is less developed (9–12). International survey data have demonstrated variation in paediatric milk and egg allergy management across countries, underscoring that implementation practices are shaped by local resources, professional norms, and service structures (13, 14).

In Australia and New Zealand, limited data describe how clinicians across disciplines and settings use food ladders, which resources they employ, and how clinical context, such as prior anaphylaxis, shapes decision-making. We therefore conducted an exploratory descriptive survey of Australasian Society of Clinical Immunology and Allergy (ASCIA)-affiliated healthcare professionals to characterise respondent-reported food ladder practices.

## Method

### Design, participants, and administration

We conducted a cross-sectional online survey of ASCIA-affiliated healthcare professionals practising in Australia and New Zealand. The 20-item questionnaire covered demographics, indications for food ladder use, implementation practices, and five scenario-based decisions involving a 9-month-old infant with milk or egg allergy across common clinical contexts. The full questionnaire is provided in the Appendix.

The survey was open from 1 June 2024 to 30 June 2024. Invitations were distributed via the ASCIA email distribution list. No reminders were sent. The number of clinicians on the distribution list was not available to the authors; therefore, the response rate could not be calculated. Participants were requested to complete the questionnaire only once. Duplicate responses were not technically prevented.

### Instrument and data handling

Items were multiple choice with optional free-text fields. No formal piloting or validation was undertaken. Respondents who reported not using food ladders selected N/A for food-ladder-specific items and were excluded from the denominator for those specific analyses.

Responses were exported to a spreadsheet. Data were anonymised and stored on secure institutional systems accessible only to study investigators.

### Statistical analysis

Analyses were descriptive by design. We report counts and percentages using valid-response denominators for each item. Respondent-level and scenario items are reported using *n* = 70 unless otherwise specified. Food-ladder-specific items are reported among food ladder users (*n* = 66). No inferential statistical testing was performed because the study was exploratory, the response denominator was unavailable, and several potential subgroup cells were small. Any subgroup observations were interpreted as hypothesis-generating only.

### Ethics

This study was conducted with reference to the National Statement on Ethical Conduct in Human Research 2023. The survey involved healthcare professionals only and did not collect patient-level data, identifiable information, or sensitive personal health information. In accordance with local institutional practice for low-risk, anonymous surveys of healthcare professionals, formal human research ethics committee review was not sought. Participation was voluntary and anonymous, and completion of the questionnaire was taken as implied consent.

## Results

### Respondents and practice settings

Respondent characteristics and implementation practices are summarised in Table 1. Seventy clinicians participated. Respondents were from Australia (45/70, 64.3%) and New Zealand (25/70, 35.7%). Disciplines included allergy/immunology specialists (29/70, 41.4%), general paediatricians (13/70, 18.6%), nurses/nurse practitioners (10/70, 14.3%), dietitians (10/70, 14.3%), trainee doctors (7/70, 10.0%), and one general practitioner (1/70, 1.4%). Most respondents primarily treated paediatric patients (53/70, 75.7%); 12/70 (17.1%) treated mixed populations and 5/70 (7.1%) primarily treated adults. Practice sectors were public (37/70, 52.9%), private (17/70, 24.3%), and both public and private (16/70, 22.9%).

**Table 1:** Respondent characteristics and implementation practices.

Characteristic	n/N (%)
Australia	45/70 (64.3)
New Zealand	25/70 (35.7)
Allergy/immunology specialist	29/70 (41.4)
General paediatrician	13/70 (18.6)
Nurse/nurse practitioner	10/70 (14.3)
Dietitian	10/70 (14.3)
Trainee doctor	7/70 (10.0)
General practitioner	1/70 (1.4)
Primarily paediatric practice	53/70 (75.7)
Public sector	37/70 (52.9)
Private sector	17/70 (24.3)
Both public and private sectors	16/70 (22.9)
Reported food ladder use	66/70 (94.3)
Use in both IgE- and non-IgE-mediated contexts^a^	47/66 (71.2)
Standardised ladder used^a^	43/66 (65.2)
Recipes provided^a^	43/66 (65.2)
Home introduction protocol provided^a^	45/66 (68.2)
Step up after multiple successful ingestions^a^	62/66 (93.9)
Reattempt non-tolerated step after 1–3 months^a^	30/66 (45.5)
Reattempt non-tolerated step after 3–6 months^a^	22/66 (33.3)
Exclusion: anaphylaxis to proposed ladder food^a^	51/66 (77.3)
Exclusion: poorly controlled asthma^a^	38/66 (57.6)

a Denominator restricted to food ladder users (*n* = 66).

Food ladder use was commonly reported among respondents (66/70, 94.3%). Among food ladder users, use was most frequently reported in both IgE- and non-IgE-mediated allergy (47/66, 71.2%), followed by IgE-mediated allergy alone (11/66, 16.7%) and non-IgE-mediated allergy alone (8/66, 12.1%).

### Food ladder types, supports, and progression advice

**Figure 1:**
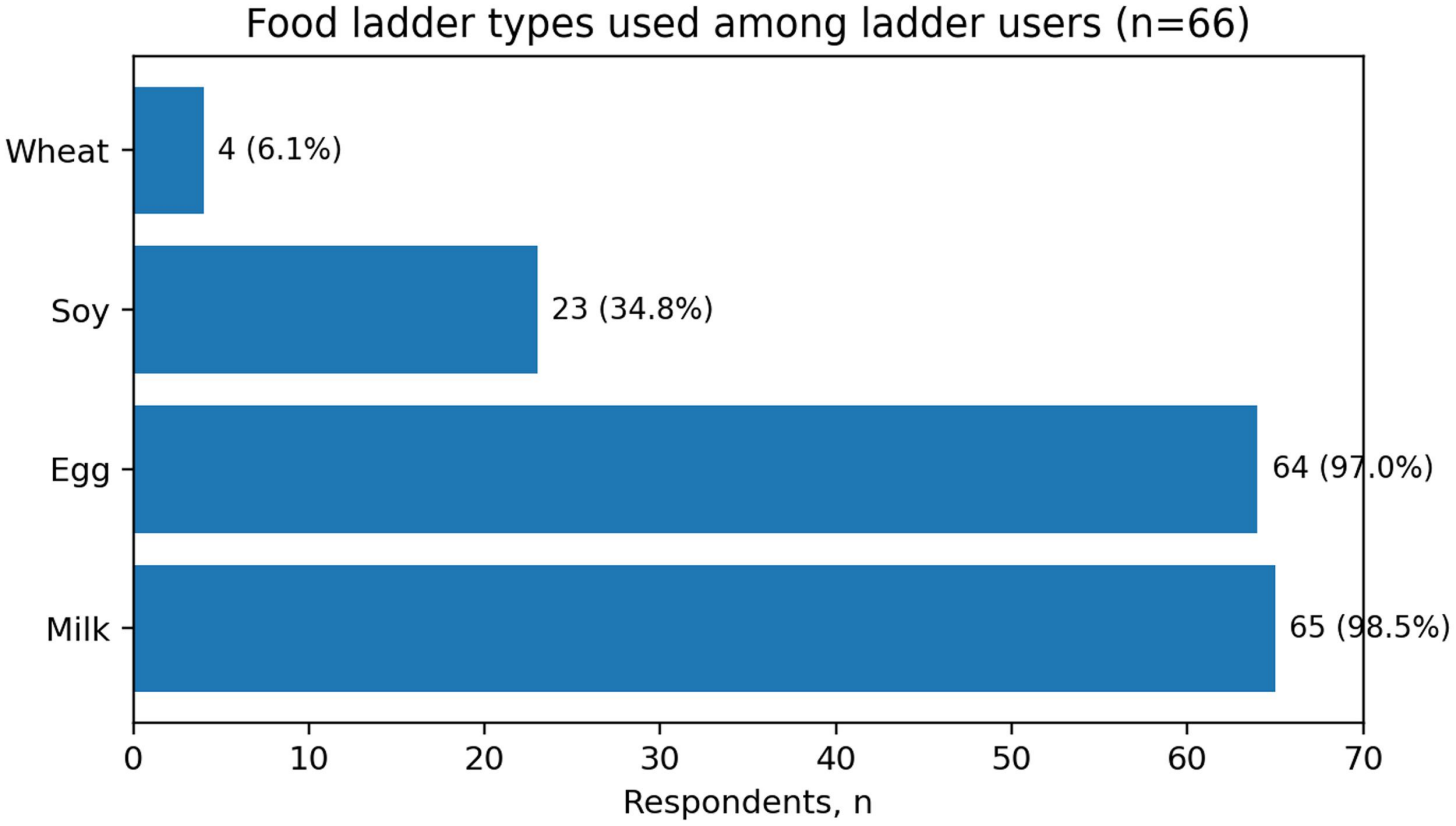
Food ladder types used among ladder users (*n* = 66).

**Figure 2:**
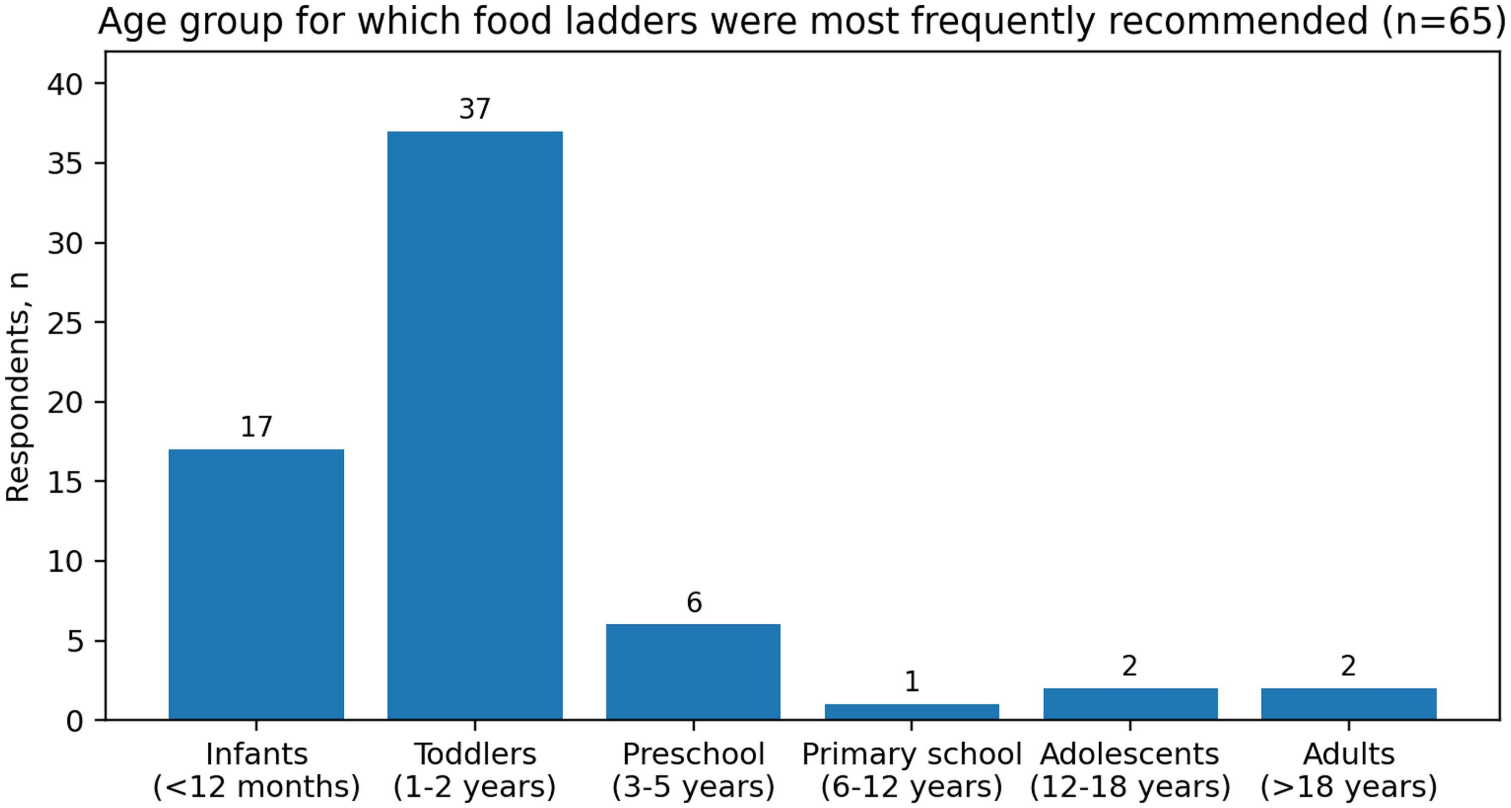
Age group for which food ladders were most frequently recommended (valid responses *n* = 65).

Milk and egg ladders predominated among food ladder users: milk 65/66 (98.5%) and egg 64/66 (97.0%). Soy ladders were reported by 23/66 (34.8%) and wheat ladders by 4/66 (6.1%) (Figure 1). The age group for which food ladders were most frequently recommended was toddlers aged 1–2 years (37/65, 56.9%), followed by infants aged <12 months (17/65, 26.2%) (Figure 2).

Standardised ladders were reported by 43/66 (65.2%) food ladder users, including iMAP, IFAN, Canadian, New Zealand national or Allergy Special Interest Group, and institution-specific ladders. Recipes and home food introduction protocols were provided by 43/66 (65.2%) and 45/66 (68.2%) respondents, respectively. Most respondents advised progression to the next ladder step after multiple successful ingestions (62/66, 93.9%). When a ladder step was not tolerated, the most commonly recommended intervals before reattempt were 1–3 months (30/66, 45.5%) and 3–6 months (22/66, 33.3%).

### Exclusions and scenario-based decisions

Sixty of 66 ladder users (90.9%) reported exclusion criteria. The most frequently cited exclusions were prior anaphylaxis to the proposed ladder food (51/66, 77.3%) and poorly controlled asthma (38/66, 57.6%). Additional free-text themes included severe FPIES, poorly controlled eczema, recent reactions to baked forms, high sensitisation, health literacy or psychosocial constraints, and active trial participation.

Scenario responses are summarised in Table 2. Respondents were least likely to recommend a ladder when the scenario included prior anaphylaxis to the relevant food: milk 9/70 (12.9%) and egg 13/70 (18.6%). Recommendation was more common in non-anaphylactic scenarios, including non-IgE-mediated milk allergy (59/70, 84.3%), mild-moderate IgE-mediated milk allergy (47/70, 67.1%), and mild-moderate IgE-mediated egg allergy (55/70, 78.6%).

**Table 2:** Scenario-based food ladder recommendations*.

Scenario	Would recommend food ladder, n/N (%)	Would not recommend food ladder, n/N (%)
9-month-old infant with non-IgE-mediated cow's milk protein allergy, growing well on milk substitute	59/70 (84.3)	11/70 (15.7)
9-month-old infant with non-anaphylactic IgE-mediated cow's milk protein allergy, growing well on milk substitute	47/70 (67.1)	23/70 (32.9)
9-month-old infant with anaphylactic cow's milk protein allergy, growing well on milk substitute	9/70 (12.9)	61/70 (87.1)
9-month-old infant with non-anaphylactic IgE-mediated egg allergy	55/70 (78.6)	15/70 (21.4)
9-month-old infant with anaphylactic egg allergy	13/70 (18.6)	57/70 (81.4)

*Note that the questionnaire captured whether respondents would recommend a ladder; it did not define supervision level, timing, or protocol intensity.

## Discussion

In this exploratory survey of ASCIA-affiliated clinicians in Australia and New Zealand, food ladder use was commonly reported among respondents, particularly for milk and egg allergy. Most ladder users reported use in both IgE- and non-IgE-mediated contexts, and many used standardised ladders, recipes, and home introduction protocols. Respondents generally advised step progression after multiple successful ingestions rather than after a single ingestion, and commonly recommended reattempting a non-tolerated step after 1–6 months. Clinical context appeared important in respondent decision-making. Prior anaphylaxis to the proposed ladder food and poorly controlled asthma were the most frequently cited exclusions, and scenario responses showed the lowest likelihood of recommending a ladder when there was prior anaphylaxis to milk or egg.

These data should be interpreted as descriptive only. They do not establish the safety, effectiveness, or appropriateness of ladder use in any specific clinical phenotype, but they indicate where clinician caution appears greatest. The findings sit within a broader international literature demonstrating variation in paediatric milk and egg allergy management. Gallagher et al. reported variation in paediatric egg and milk allergy management across Ireland, Spain, and Canada, reflecting differences in local practice structures and therapeutic approaches (13). Our data provide a complementary Australasian snapshot, suggesting that variation also exists in implementation supports, perceived exclusions, and responses to higher-risk scenarios. However, because our survey denominator was unavailable and subgroup numbers were modest, these observations should not be interpreted as evidence of true geographic or professional differences.

Terminology is important. Food ladders are best understood here as graded dietary advancement tools, not as oral immunotherapy protocols (15). Although both involve exposure to allergenic food, oral immunotherapy generally involves structured dosing, planned escalation, and different monitoring and risk considerations. Similarly, ladder use should not be assumed to induce tolerance. A more cautious interpretation is that food ladders may support dietary expansion or reintroduction in selected patients under clinician guidance.

Interpretation also requires attention to allergen and clinical phenotype. Milk and egg ladders are more established in published clinical resources than soy and wheat ladders, so reported soy or wheat ladder use in this survey should be read as a description of practice rather than evidence that these approaches are equally standardised or equally supported (16, 17). The survey also did not distinguish between different non-IgE-mediated phenotypes. In particular, FPIES has distinct clinical features, including delayed symptom onset and potential for severe reactions, and reintroduction decisions require phenotype-specific risk assessment (18). Therefore, these data should not be extrapolated to all non-IgE-mediated food allergy presentations.

Strengths of this study include multinational participation across Australia and New Zealand, multidisciplinary representation, and reporting of practical implementation details such as standardised ladders, recipes, home protocols, step progression, and reattempt timing. The study also captures clinician responses to common clinical scenarios, allowing cautious description of where reported practice converges or diverges.

Regarding limitations, the survey denominator was unavailable, and the response rate could not be calculated. Selection and volunteer bias are likely, particularly as clinicians with an interest in food allergy or food ladders may have been more likely to respond. The study relied on clinician self-report and did not assess patient-level outcomes, safety events, adherence, family experience, quality of life, or objective predictors of successful dietary advancement. The questionnaire was not formally validated, and duplicate responses were not technically prevented. Subgroup analyses were limited by small denominators and were not subjected to inferential testing.

Future work should prospectively evaluate ladder-guided food reintroduction practices in clearly defined clinical phenotypes and allergens, with attention to safety, adherence, family experience, and clinical outcomes. Consensus terminology and implementation resources may help reduce unwarranted variation, and their development should be informed by prospective, high-quality evidence and multidisciplinary input.

## Conclusion

This exploratory survey describes respondent-reported food ladder practices among ASCIA-affiliated clinicians in Australia and New Zealand (19). Food ladder use was commonly reported among respondents, particularly for milk and egg allergy, with variability in implementation supports and responses to higher-risk clinical contexts such as prior anaphylaxis. These findings are descriptive and hypothesis-generating, and support further prospective evaluation and consensus-based standardisation.

## Data Availability

The original contributions presented in the study are included in the article/Supplementary Material, further inquiries can be directed to the corresponding author.

## Appendix

QUESTIONNAIRE

Thank-you for taking the time to complete this questionnaire. We kindly request that you participate in this

survey if you are an ASCIA-affiliated healthcare professional. Your insights are invaluable to our

research. The questionnaire will take approximately 5 min to complete.

1. Are you an ASCIA-associated healthcare professional?

a. Yes

b. No

2. What is your professional background?

a. Allergy/immunology specialist

b. General practitioner

c. Trainee doctor

d. Dietitian

e. Nurse/nurse practitioner

f. Other

2a. If you answered “Other” in question 2, please specify your professional background. Or, answer “N/A.”

3. Which sector do you work in?

a. Private

b. Public

c. Both

4. Which state or territory do you practise in?

a. Australian Capital Territory

b. New South Wales

c. Northern Territory

d. Queensland

e. South Australia

f. Tasmania

g. Victoria

h. Western Australia

i. New Zealand

5. Which population do you primarily work with?

a. Paediatric (≤18 years of age)

b. Adult (>18 years of age)

c. Mixed

6. Do you use food ladders in your practice?

a. Yes

b. No

7. Which type of allergy do you use food ladders for?

a. IgE-mediated

b. Non-IgE mediated

c. Both

d. N/A

8. Which of the following ladders do you use in your clinical practice (please tick all that apply)?

a. Milk

b. Egg

c. Soy

d. Wheat

e. Other

f. N/A

8a. If you answered “Other” in question 8, please specify the food ladder. Or, answer “N/A.”

9. Do you use standardised ladders (e.g., iMAP milk ladder, IFAN egg ladder, etc.)?

a. Yes

b. No

c. N/A

9a. If you answered “Other” in question 9, please specify what kind of standardised ladder you provide.

Or, answer “N/A.”

10. Do you provide recipes to assist with food ladders?

a. Yes

b. No

c. N/A

11. Do you provide a home food introduction protocol to assist with food ladders?

a. Yes

b. No

c. N/A

12. If a patient tolerates a ladder step, when do you advise to climb up the ladder?

a. After single ingestion

b. After multiple ingestions

c. Other

d. N/A

12a. If you answered “Other” in question 12, please specify what frequency. Or, answer “N/A.”

13. If a patient does not tolerate a ladder step, when do you advise to reattempt the same step?

a. 1–3 months

b. 3–6 months

c. 6–12 months

d. Other

e. N/A

13a. If you answered “Other” in question 13, please specify the length of time. Or, answer “N/A.”

14. Do you have exclusion criteria that precludes a patient from utilising food ladders?

a. Yes

b. No

c. N/A

14a. If so, what are they (please select all that apply)?

a. Asthma

b. Poorly controlled asthma

c. Anaphylaxis to proposed ladder food

d. Anaphylaxis to any food

e. Other

f. “N/A”

14b. If you answered “Other” in question 14a, please specify. Or, answer “N/A.”

15. For which age group do you most frequently recommend food ladders?

a. Infants (<12 months)

b. Toddlers (1–2 years)

c. Preschool (3–5 years)

d. Primary school (6–12 years)

e. Adolescents (12–18 years)

f. Adults (>18 years of age)

g. N/A

16. 9-month-old infant with non-IgE mediated cow's milk protein allergy. Growing well on a milk

substitute. Would you recommend a milk ladder?

a. Yes

b. No

c. N/A

17. 9-month-old infant with non-anaphylactic IgE-mediated cow's milk protein allergy. Growing well on a

milk substitute. Would you recommend a milk ladder?

a. Yes

b. No

c. N/A

18. 9-month-old infant with anaphylactic cow's milk protein allergy. Growing well on milk substitute. Would

you recommend a milk ladder?

a. Yes

b. No

c. N/A

19. 9-month-old infant with non-anaphylactic IgE-mediated egg allergy. Would you recommend an egg

ladder?

a. Yes

b. No

c. N/A

20. 9-month-old infant with anaphylactic egg protein allergy. Would you recommend an egg ladder?

a. Yes

b. No

c. N/A

Thank-you for taking the time to complete this questionnaire. Your input is greatly appreciated and will

significantly contribute to our understanding of food ladder utilisation practices among ASCIA healthcare

professionals. We look forward to sharing our findings at an upcoming local conference and contributing

to the advancement of best practices in allergy management.
